# Optimization of ski jumping in-run posture using computational fluid dynamics

**DOI:** 10.1038/s41598-025-00710-2

**Published:** 2025-07-16

**Authors:** Wenhan Liu, Feixiang Lu, Xiang Suo, Weidi Tang

**Affiliations:** 1 School of Intelligent Sports Technologies, Shanghai University of Sport, Shanghai, 200438 China; 2 SUS-Baidu PaddlePaddle Intelligent Sports Technology Innovation Center, Shanghai, 200438 China

**Keywords:** Computational fluid dynamics, Ski jumping, In-run stage, Aerodynamic characteristics, Posture optimization, Biomedical engineering, Computational science

## Abstract

While aerodynamic optimization during ski jumping flight phases is well-studied, critical knowledge gaps persist regarding posture-fluid interactions in the in-run phase – particularly the dominance of drag dynamics over lift enhancement for speed maximization. This study establishes an athlete-specific 3D model to investigate posture-dependent resistance through high-resolution CFD simulations. Systematically analyzing four key posture parameters – torso attack angle (α), thigh attack angle (β), ankle joint angle (γ), and hip abduction angle (ε) – reveals α as the governing factor influencing aerodynamic resistance during acceleration. The optimized configuration ($\:\alpha\:\in\:\left[0^\circ\:,2^\circ\:\right]$, $\:\beta\:\in\:\left[20^\circ\:,22^\circ\:\right]$, $\:\gamma\:\in\:\left[43^\circ\:,45^\circ\:\right]$, and $\:\epsilon\:\in\:\left[-2^\circ\:,0^\circ\:\right]$) reduces cumulative air resistance by approximately 5% compared to conventional postures, demonstrating that marginal angular adjustments in torso positioning significantly outweigh other joints’ contributions to drag reduction. Contrary to flight-phase strategies emphasizing lift generation, the results establish drag minimization as the primary in-run optimization objective. These findings provide evidence-based posture guidelines for athletes while advancing a paradigm shift from empirical to physics-driven training methodologies – particularly through computational fluid dynamics with practical sports biomechanics. The work positions CFD as an indispensable tool for quantifying millimeter-scale posture adaptations in winter sports equipment-athlete system optimization.

## Introduction

Ski jumping is a captivating winter sport that combines athleticism, technique, and mental fortitude. The sport has evolved significantly since its inception, with advancements in equipment, training methods, and venue design contributing to the remarkable distances and heights achieved by modern ski jumpers. The in-run phase, where athletes gain speed before takeoff, is crucial in determining the trajectory and velocity of the jumper, directly impacting the distance and style of the jump^1,2^. Research confirms in-run speed critically influences flight distance, as higher takeoff velocity boosts kinetic energy and lift. However, exact gains depend on hill geometry, aerodynamics, posture optimization^3^. However, the complexity of fluid dynamics involved makes it challenging to identify the optimal posture for each individual athlete. Computational Fluid Dynamics (CFD) has emerged as a transformative tool in sports biomechanics for resolving posture- and equipment-related aerodynamic challenges, including investigate air resistance in cycling^4^, analyze swimmer wake flows^5^ and optimize speed skating posture^6^. In ski jumping, CFD studies have predominantly focused on the flight phase, analyzing jumper-ski interactions and lift-to-drag ratios, and enabling rapid evaluation (e.g., 50 + posture/equipment variations within hours) of design parameters such as ski geometry or posture adjustments^7,8^. Furthermore, CFD simulations provide detailed insights into the flow field, helping athletes and coaches understand the underlying mechanisms of performance enhancement.

Despite the challenges in accurate modeling of athlete geometry and motion^9,10^ and selection of appropriate turbulence models and boundary conditions^11,12^, the use of CFD simulation in sports biomechanics is expected to grow rapidly. This study utilizes high-resolution CFD simulations and an athlete-specific model to systematically analyze in-run aerodynamic resistance, providing a physics-driven approach to posture optimization and demonstrating CFD’s potential as an efficient alternative to traditional experimental methods, with potential implications for ski jumping training, performance, and the broader field of sports biomechanics.

## Methods

### Mathematical modeling

**Fig. 1:**
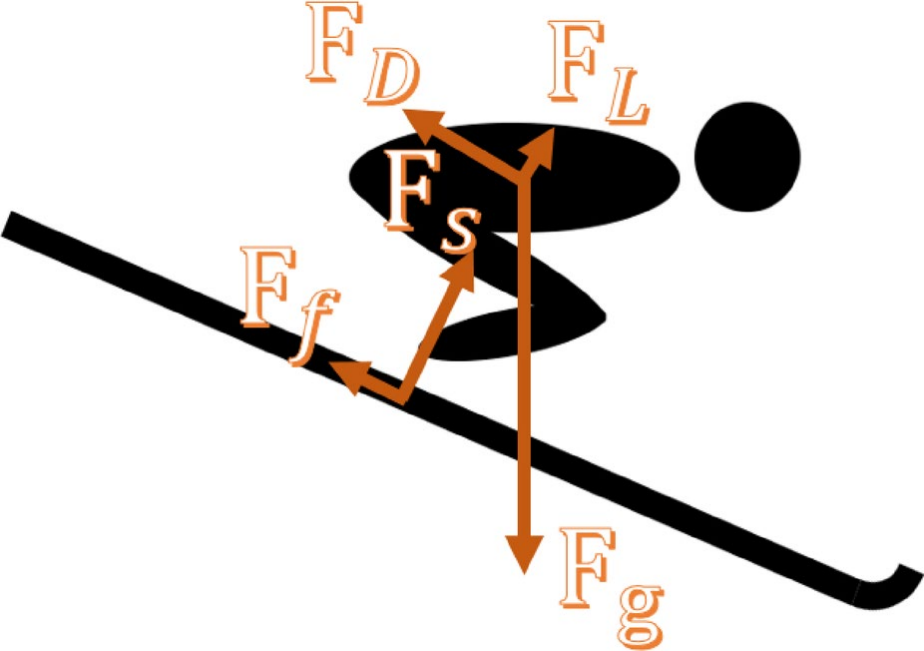
Analysis of the force during the athlete’s in-run phase.

The in-run phase takes place entirely on the in-run track. According to the International Ski Federation’s regulations on the construction of ski jumping hills, the in-run track is designed to consist of a long straight section, a curved section with a radius of approximately 150 m, and a final short straight section(Some hills use parabolic transitions, though our model assumes a constant radius for simplicity)^13^. During this process, athletes are mainly subjected to four forces: gravity $\:{F}_{g}$, air resistance $\:{F}_{D}$, sliding friction $\:{F}_{f}$ (which includes the friction between the bottom of the skis and the ice surface as well as the friction between the sides of the skis and the grooves of the track), and support force from the track $\:{F}_{s}$. (See Fig. 1) Athletes maintain a low crouched position throughout the in-run phase, with the torso and the in-run track forming a parallel or nearly parallel angle. These forces, such as lift and drag, affect the jumper’s speed and trajectory, ultimately determining the success of the jump. Therefore, optimization of the athlete’s posture during the in-run phase is essential for enhancing the performance and safety of ski jumpers.

$\:{F}_{g}$ is the force of gravity acting on the system and points vertically downward; $\:{F}_{D}$ is the force of air resistance acting on the system and points in the opposite direction to the system’s motion; $\:{F}_{f}$ is the force of sliding friction acting on the system and points in the opposite direction to the system’s motion; $\:{F}_{s}$ is the support force acting on the system and is perpendicular to the surface of the track. $\:{F}_{L}$ is the force of lift or upward force acting on the system, caused by its motion through the air, and it is perpendicular to the direction of motion, pointing upwards.

To simplify the analysis process, the system is considered as a rigid body, an assumption that has been employed in prior studies^14^ and the sliding friction and weight acting on the system (where $F_{g} = m \cdot g$, with m being the total mass of the system and g being the gravitational acceleration, usually considered constant) are assumed to be independent of the athlete’s in-run posture. Therefore, the variables in the system that are affected by the athlete’s posture during the in-run phase are the air resistance $\:{F}_{D}$ and lift forces $\:{F}_{L}$ acting on the system. The aerodynamic drag and lift forces (Eqs. 1 and 2) are derived based on the standard aerodynamic equations and calibrated using empirical coefficients from Virmavirta’s ski-jumping-specific fluid dynamics framework^2^. Equations (1) and (2), originally formulated in the Aquila simulator’s aerodynamic module:

1 $$\:{F}_{D}=\frac{1}{2}\rho\:{v}^{2}{C}_{D}A$$

2 $$\:{F}_{L}=\frac{1}{2}\rho\:{v}^{2}{C}_{L}A$$

Here, $\:\rho\:$ is the air density; A is the cross-sectional area of the system; $\:{C}_{D}$ is the drag coefficient of the system; $\:{C}_{L}$ is the lift coefficient of the system; and $\:v$ is the speed of the system. These parameters are all related to the aerodynamic and physical properties, which significantly affect the in-run and flight performance of ski jumping athletes.

The in-run speed $\:v$ appears as the only quadratic term in the equations, with the largest weight in the output value. This indicates that the in-run speed is the primary factor affecting the air resistance and lift forces acting on the system. Additionally, the square of the in-run speed is directly proportional to the air resistance and lift forces acting on the system. Other variables such as the lift and drag coefficients are determined by the aerodynamic shape and materials of the system and are relatively stable. The remaining factor that is directly related to the athlete’s posture is the surface area of the system. In the study of air resistance $\:{F}_{D}$, the effective surface area A refers to the projected area of the system on the plane perpendicular to the direction of motion. The goal of techniques such as lowering the upper body to the horizontal plane is to reduce the value of $\:{C}_{D}*A$ for the system. In the 2006 Turin Winter Olympics, Mikko Virmavirta’s image analysis revealed that in-run speed exhibited the strongest correlation with jump distance, surpassing other measured variables, including hip angular velocity in top performers^15^. However, optimizing the in-run posture to meet both the athlete’s jumping force requirements and reduce the wind-facing area of the system remains an unanswered question.

In this study, a 3D athlete-specific model of the athlete/skis multi-body system was established to seek the optimal aerodynamic posture during the in-run stage of ski jumping, focusing on dominant factors such as the attack angle of the torso and thighs and their effects on the overall aerodynamic characteristics of the system^16^. Computational fluid dynamics analysis was conducted on the system in a simulated in-run environment to obtain the aerodynamic characteristics of different in-run postures and the lift and drag forces acting on the system during the in-run phase. The optimal limb angles of the athlete-specific model multi-body system were integrated under different posture angles, and the aerodynamic characteristics of the athlete’s specific in-run posture were explored at different speeds. An aerodynamic model was used to quantify the in-run posture and optimize it to obtain better jumping conditions, ultimately helping the athlete achieve a farther flying distance.

### CFD simulation setup

This article studies the multi-body system consisting of ski jumpers’ body, skis, helmet, and other equipment. The body posture features of the model were based on the body measurement data of male athletes of China in the ski jumping team during the 2022 training camp, as shown in Table 1.

**Table 1:** Anthropometric measurements of individual athletes (A–E) and the derived mean across all subjects. Units are provided for each measurement category.

Measurement items	A	B	C	D	E	Derived mean
Height/cm	173	178	177	170	177	175
Weight/kg	54	59	65	54.5	56.5	57.8
Neck circumference/cm	37	36	37.5	36	36	36.5
Chest circumference/cm	90	87	96	87	84	88.8
Waist circumference/cm	74	70	76	71	73	72.8
Abdominal circumference/cm	80	81	79	76	86	80.4
Pants waist circumference/cm	71	70	75	69	71	71.2
Hip circumference/cm	87	89	92	86	91	89
Thigh circumference/cm	52	57	55	52	53	53.8
Calf circumference/cm	34	34	39	33	32	34.4
Crotch depth/cm	69	67	65	70	71	68.4
Upper arm circumference/cm	29	30	30	28	27	28.8
Wrist circumference/cm	15	16	17	16	15	15.8
Shoulder width/cm	46	41	47	45	45	44.8
Sleeve length/cm	63	63	64	60	60	62
Pant length/cm	104	100	98	98	99	99.8
Ankle circumference/cm	21	22	24	20	21	21.6

**Fig. 2:**
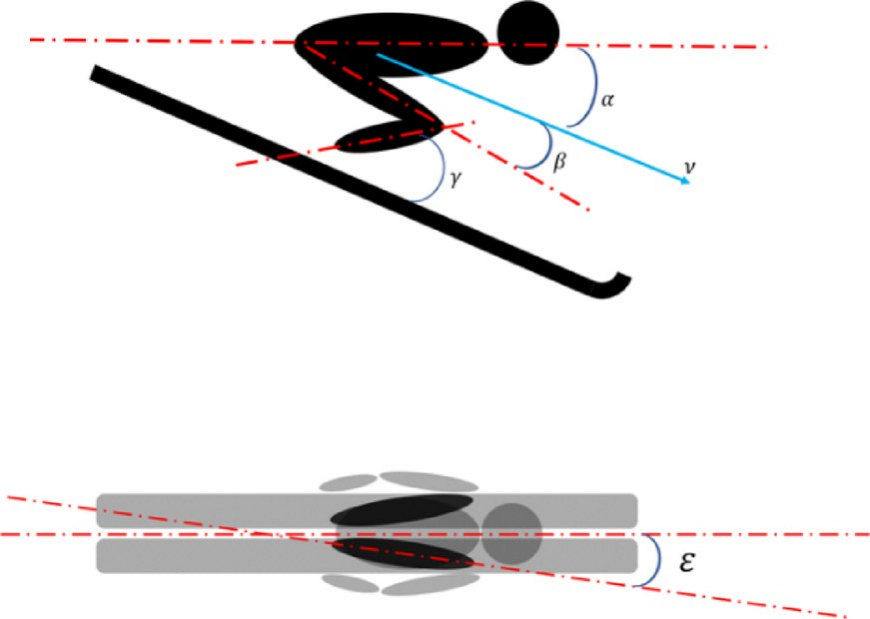
Position parameters of the in-run phase. $\:\overrightarrow{\nu\:}$: in-run speed, $\:\alpha\:$: angle of attack, $\:\beta\:$:Thigh angle of attack, $\:\gamma\:$: ankle joint angle, $\:\epsilon\:$: hip joint abduction angle

During the in-run stage, ski jumpers’ kinematic parameters, including in-run speed $\:\overrightarrow{\nu\:}$, angle of attack $\:\alpha\:$, Thigh angle of attack $\:\beta\:$, ankle joint angle $\:\gamma\:$, hip joint abduction angle $\:\epsilon\:$, as shown in Fig. 2. The positive directions of these angles are indicated in the figure.

### Turbulence models and boundary conditions

#### Turbulence model

The k-ω turbulence model is so far the most widely used model in fluid turbulence research, and its effectiveness has been verified in various applications in different fields^17^. In this research, Shear-Stress Transport(SST) k-ω Model is used. The SST k-ω model includes all improvements of the baseline k-ω model, which is a two-equation model for turbulence that aims to capture the effects of both Reynolds stress and shear stress. The model was developed to address the limitations of the standard k-ω model, which does not account for shear stress effects accurately. The SST k-ω model incorporates an additional term to account for shear stress effects and introduces a supplementary transport equation for turbulent viscosity. Its accuracy has been validated in high-speed sports aerodynamics through cross-validation with wind tunnel data^18,19^. This improves the accuracy and reliability of the model. Its expression is as follows:

3 $$\:\frac{\partial\:\left(\rho\:k\right)}{\partial\:t}+\frac{\partial\:\left(\rho\:k{u}_{i}\right)}{\partial\:{x}_{i}}={P}_{k}-\rho\:{\beta\:}^{*}k\omega\:+\frac{\partial\:}{\partial\:{x}_{i}}\left[\left(v+{\sigma\:}_{k}\frac{{v}_{t}}{{\sigma\:}_{\omega\:}}\right)\frac{\partial\:k}{\partial\:{x}_{i}}\right]$$

4 $$\:\frac{\partial\:\left(\rho\:\omega\:\right)}{\partial\:t}+\frac{\partial\:\left(\rho\:\omega\:{u}_{i}\right)}{\partial\:{x}_{i}}=\frac{{\gamma\:}^{*}}{\omega\:}\left({\alpha\:}^{*}{P}_{k}-\rho\:{\beta\:}^{*}{\omega\:}^{2}\right)+\frac{\partial\:}{\partial\:{x}_{i}}\left[\right(v+{\sigma\:}_{\omega\:}\frac{{v}_{t}}{{\sigma\:}_{\omega\:}}\left)\frac{\partial\:\omega\:}{\partial\:{x}_{i}}\right]$$

where $\:\rho\:$ is the fluid density, $\:{u}_{i}$represents the velocity components, $\:v$ is the fluid kinematic viscosity, $\:k$ is the turbulent kinetic energy, $\:\omega\:$ is the specific dissipation rate, $\:{P}_{k}$​ represents the production of turbulent kinetic energy, and $\:{\gamma\:}^{*}$, $\:{\alpha\:}^{\text{*}}$, $\:{\beta\:}^{\text{*}}$, $\:{\sigma\:}_{k}$, and $\:{\sigma\:}_{\omega\:}$ are model constants.

#### Computational geometry, domain, and grid

Based on body measurement data and in-run posture parameters, a three-dimensional athlete-specific model of ski jumping athletes in the in-run stage is established, and the system is modeled in 3D. Joint anchor points are set in the model to enable accurate control of the model’s motion angles, in order to meet the requirements of different joint angles in the research.

**Fig. 3:**
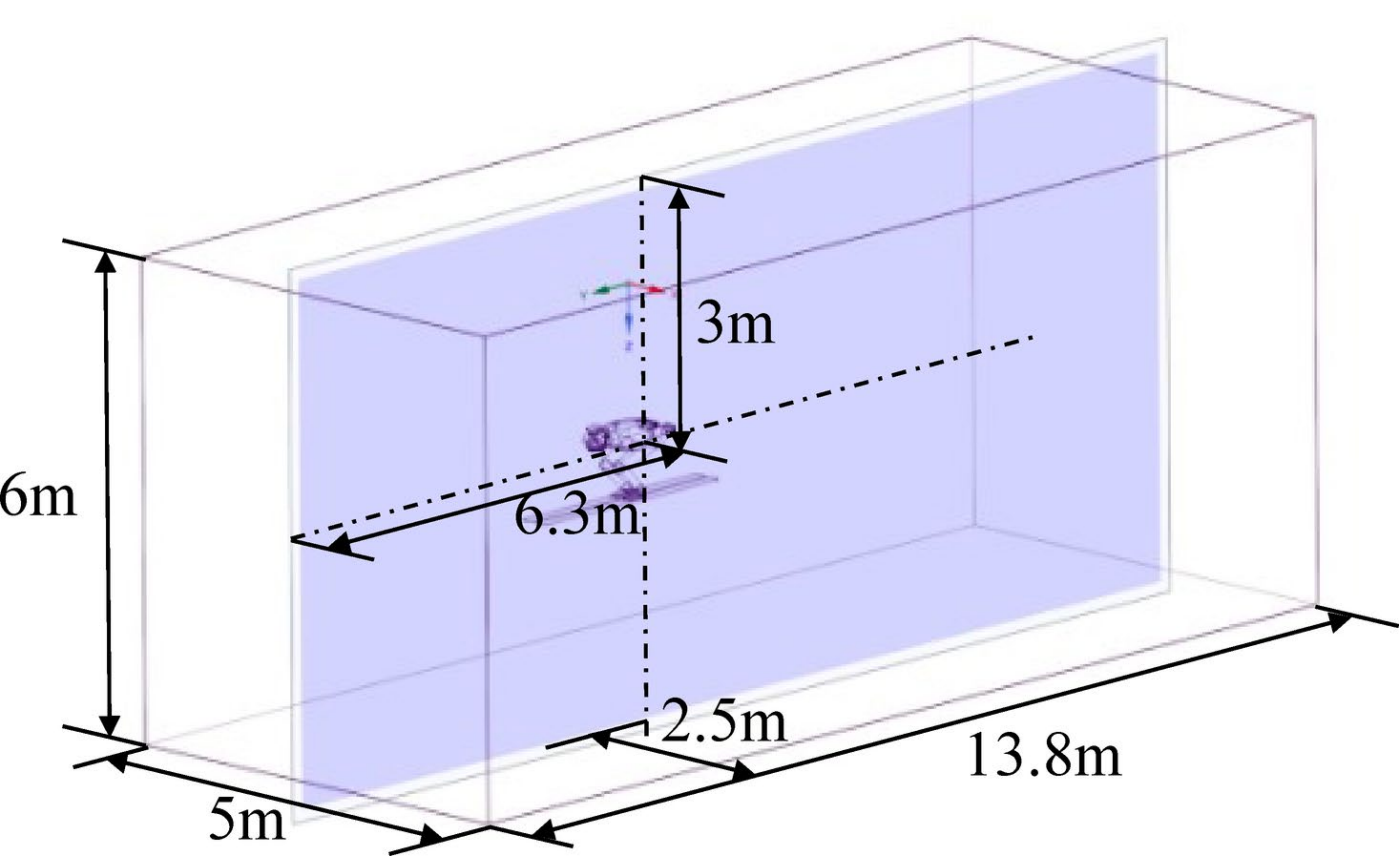
Schematic diagram of flow field and multi-body model.

In this study, the range of the fluid domain is set to 6 m × 5 m × 13.8 m^20^, and the model of the ski jumper is located at the center of the normal direction of the pressure inlet surface of the flow field, 6.3 m away from the inlet surface, as shown in Fig. 3.

The flow field can be divided into several regions, including the inlet surface where the fluid velocity enters the domain, the outlet surface where the fluid velocity exits the domain, the target surface which represents the model surface, the symmetry surface which represents the center plane of symmetry, and the domain surface which represents the domain boundary. Based on the related research of simulating and calculating the aerodynamic characteristics of ski jumping hill^21^, In CFD modeling and simulation of ski jumping flow field, the optimal minimum grid resolution is 0.5 mm. The finer grid cells are mainly distributed near the numerical twin model of the athlete to accurately simulate the small changes in flow field around the model. The larger grid cells are distributed away from the model to reduce the computational cost without affecting the calculation results. In the refined CFD simulation of the model, the number of polyhedrons on the model surface is around 40 layers with a growth rate not greater than 1.1^19^.

**Table 2:** Results of grid-independency test.

	Discrete scheme 1	Discrete scheme 2	Discrete scheme 3	Discrete scheme 4
Total Grid(million)	10	14.66	19.87	28.38
Lift-to-drag	1.949	1.948	1.951	1.949

**Fig. 4:**
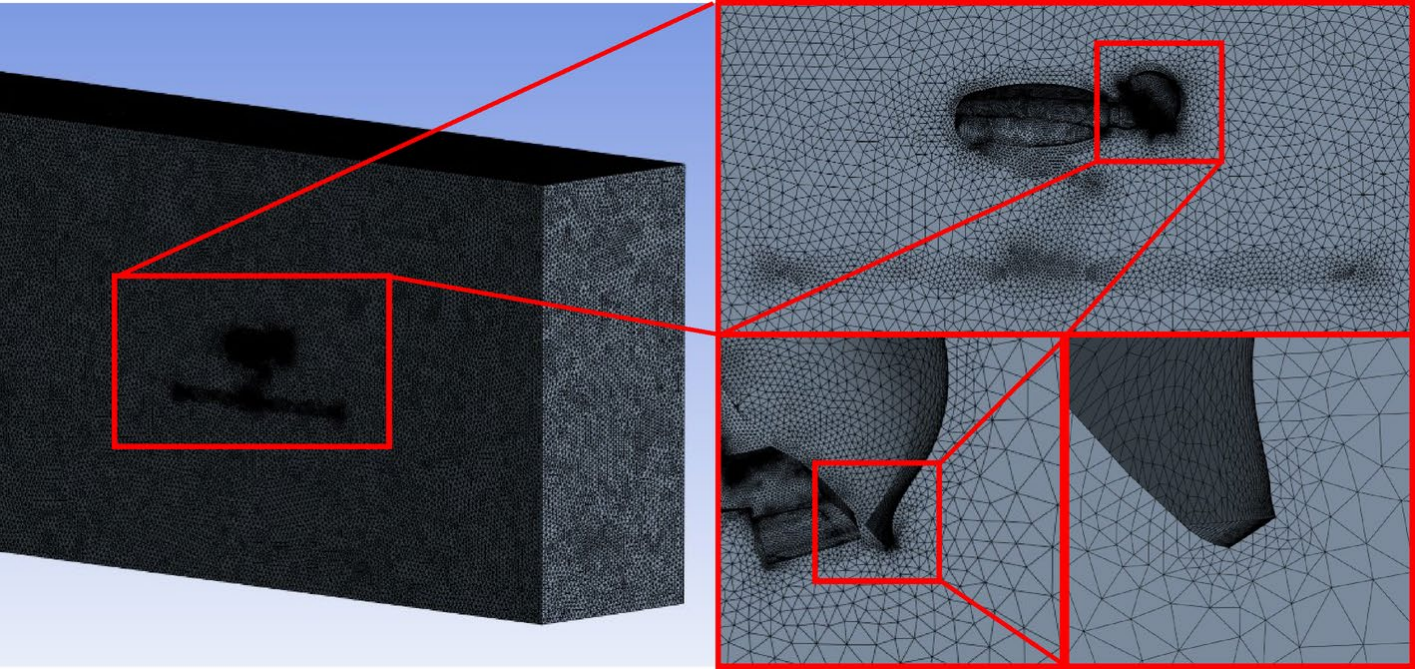
Model grid distribution.

Through the grid-independency test, as shown in Table 2, the variation of grid numbers in the range of $\:1.0\times\:{10}^{7}\sim{2.5\times\:10}^{7}$ did not have a significant impact on the accuracy of the model^22^, therefore, in this study, a refined mesh division method was adopted with an actual grid number of 23,628,556. Figure 4 shows the mesh division of the model.

Boundary conditions define the behavior of the fluid at the boundaries of the computational domain, and they play a critical role in determining the accuracy of the simulation results. In the case of the ski jumping flow field, the inlet boundary conditions are set as a constant velocity profile which represents the skier’s initial speed. According to the research conclusion on the aerodynamics in the in-run stage of ski jumping, the end speed is set at 25 $\:m/s$[18]. The flow is assumed to be turbulent, with inlet turbulence characteristics based on experiments or theory. Zero-pressure outlet conditions are applied, assuming fully developed flow and no significant streamwise pressure gradient, valid when the outlet is far downstream^23^. This implies no backflow at the outlet. The no-slip condition is set at the walls, with zero velocity, following fundamental fluid dynamics principles for viscous flows^24^. The symmetry boundary conditions are applied to the center plane of the ski jumping hill, which assumes that the flow is symmetric with respect to this plane. In wind tunnel tests and numerical simulations for the ski jumping in-run phase, Elfmark et al. found that there is an optimal range for posture parameters, such as the pitch angle of the torso and the Thigh angle of attack. However, the optimal combination of these parameters was not identified through numerical analysis^1^. According to the research, the optimal range for the pitch angle of the torso $\:\alpha\:$ is: $\:0^\circ\:\sim4^\circ\:$, The optimal range for the Thigh angle of attack $\:\beta\:$ is: $\:22^\circ\:\sim26^\circ\:$, The optimal range for the ankle joint angle $\:\gamma\:$ is: $\:45^\circ\:\sim49^\circ\:$. The optimal range for the hip joint abduction angle $\:\epsilon\:$ is:$\:-2^\circ\:\sim2^\circ\:$. Based on the interval division, this study divided each closed interval into three discrete values and calculated all possible posture combinations. The specific velocity and posture parameters are listed in Table 3.

**Table 3:** Position parameter of CFD model.

$\:\alpha\:$	$\:\beta\:$	$\:\gamma\:$	$\:\epsilon\:$
$\:0^\circ\:$	$\:22^\circ\:$	$\:45^\circ\:$	$\:0^\circ\:$
$\:2^\circ\:$	$\:24^\circ\:$	$\:47^\circ\:$	$\:2^\circ\:$
$\:4^\circ\:$	$\:26^\circ\:$	$\:49^\circ\:$	$\:-2^\circ\:$

$\:\alpha\:$: angle of attack, $\:\beta\:$:Thigh angle of attack,

$\:\gamma\:$: ankle joint angle, $\:\epsilon\:$: hip joint abduction angle

## Results

### Overview of results

Details of different posture data for each group and the corresponding windward area ($\:{C}_{D}A$) under different posture angle combinations, system total lift, system total drag, and lift-to-drag ratio (the ratio of the total lift to drag force that the system experiences in the fluid) can be found in Table 4. Using the angles of the group A ($\:\alpha\:=0^\circ\:,\beta\:=22^\circ\:,\gamma\:=45^\circ\:,\epsilon\:=0^\circ\:$) as a baseline. The flow analysis reveals a high-pressure region at the front of the multi-body structure (helmet), followed by a low-pressure area as the fluid accelerates along the surface. A low-pressure wake forms behind this area as the fluid separates from the system at the shoulders and arms. The surface separation effect causes a slight increase in static pressure at the tail. The athlete’s torso and calves exhibit relatively low static pressure, indicating a smaller contribution to the system’s air resistance.

**Table 4:** Excerpts from fluid dynamics simulation results under different positions.

$\:\alpha\:$	$\:\beta\:$	$\:\gamma\:$	$\:\epsilon\:$	Groups	Windward area ($\:{m}^{2}$)	Drag force (*N*)	Lift area ($\:{m}^{2}$)	Lift force (*N*)	lift-to-drag ratio
0	22	45	0	Group A	0.23494	30.54704	0.92468	6.03852	0.19768
0	22	45	2	Group B	0.23816	31.09581	0.92467	7.90824	0.25432
0	22	45	-2	Group C	0.23707	30.45540	0.92467	5.55640	0.18244
0	22	45	-4	Group D	0.23144	33.56088	0.92467	5.79355	0.17263
0	22	47	0	Group E	0.23731	32.33494	0.92574	6.79802	0.21024
0	22	49	0	Group F	0.24035	32.48504	0.92470	8.92354	0.27470
0	24	45	0	Group G	0.24047	30.11432	0.92579	7.59590	0.25224
0	26	45	0	Group H	0.24589	31.54897	0.92465	4.44324	0.14084
2	22	45	0	Group I	0.23996	30.42188	0.92471	5.73027	0.18836
4	22	45	0	Group J	0.24495	32.19710	0.92360	9.96331	0.30945

Studies investigating the coefficient of friction in ski jumping have indicated that a friction coefficient of $\:\mu\:=0.04$ between the surface of the track and the skis provides a more accurate representation of the system mechanics^25,26^. Since the lift force direction is perpendicular to the velocity direction, an increase in lift force leads to a decrease in sliding support force, resulting in a decrease in frictional force. While our results suggest that lift forces may contribute to reducing in-run resistance, the observed lift-induced drag reduction effect remains limited relative to total system drag. However, conclusive validation of this relationship requires dynamic simulations of the in-run phase to directly quantify speed outcomes. This highlights the importance of calculating the influence of air resistance on the system. Therefore, in the subsequent comparative experiments, significance should be placed on calculating air resistance $\:{F}_{D\:}$ as it is the primary factor affecting the system’s performance.

**Fig. 5:**
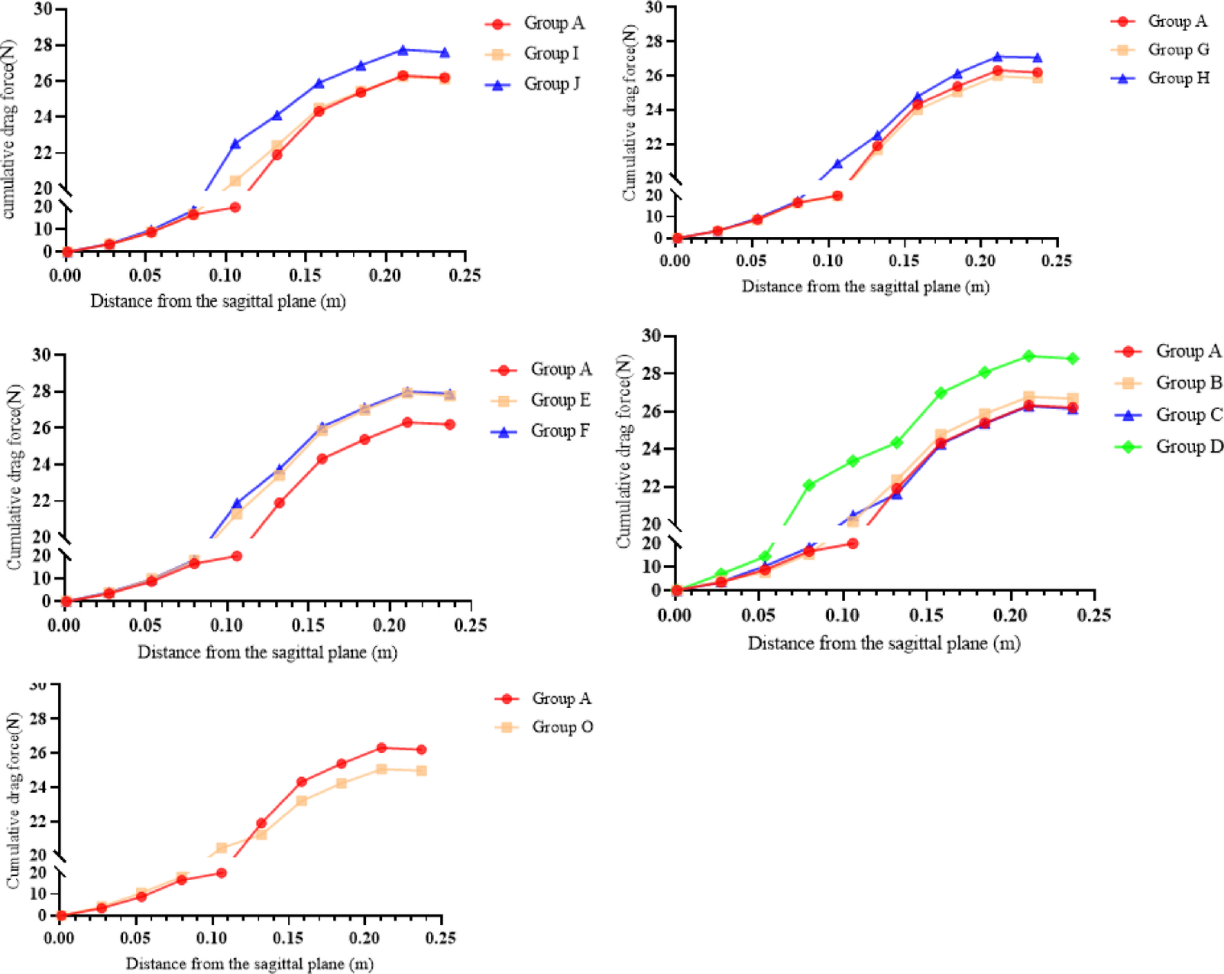
The cumulative force of the system in different groups.

As illustrated in Fig. 5 of group A, I and J, there exists a local optimal solution for the torso attack angle α at $\:\alpha\:=2$, where the cumulative air resistance experienced by the system is slightly lesser than that of the control group. Compared to $\:\alpha\:=4^\circ\:$, the reduction in air resistance is about 5.5%.

**Fig. 6:**
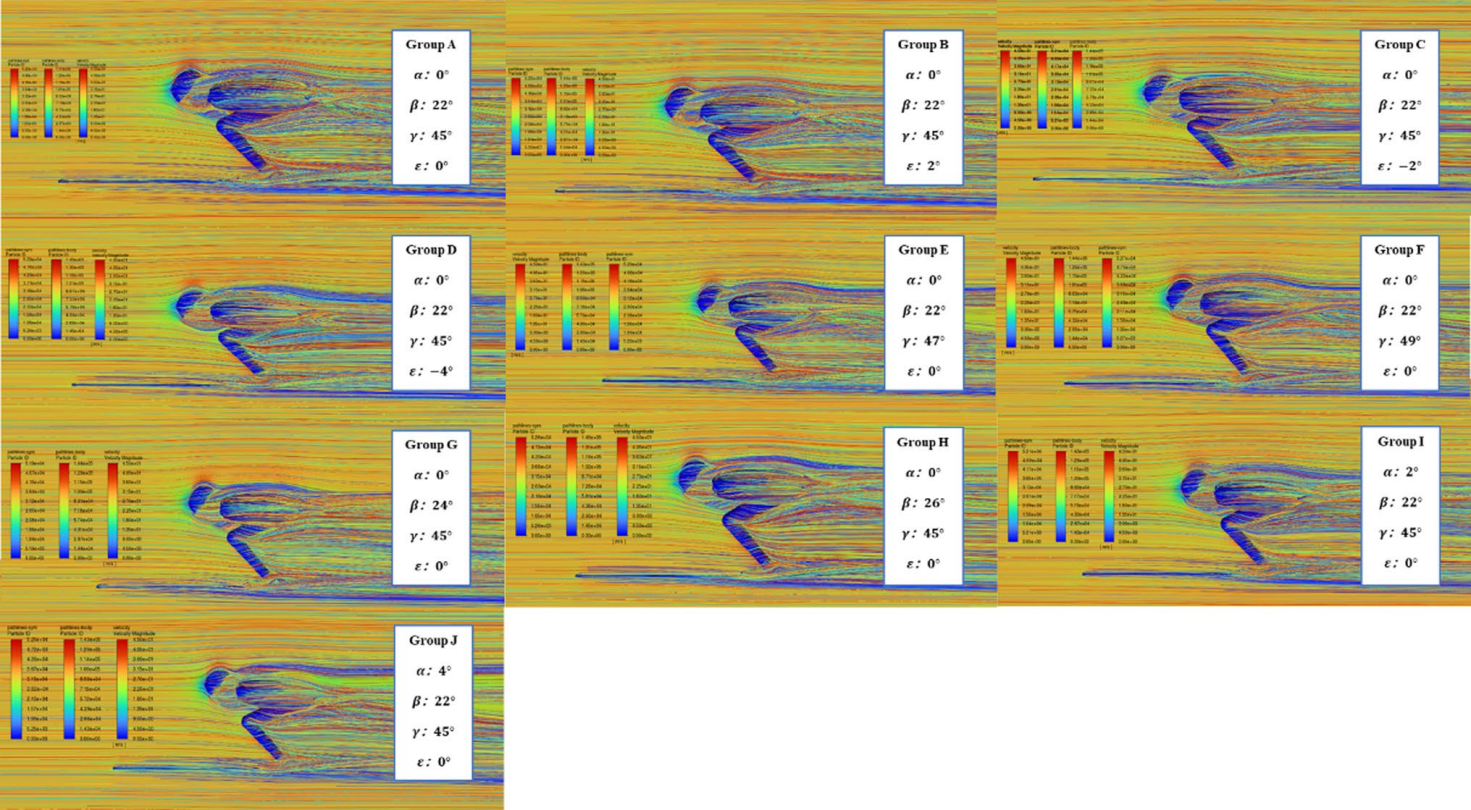
Pathlines of Each Groups, Calculated in Ansys 2021 R1 (https://www.ansys.com).

Figure 6 shows that in Group J, there are more green parts in the flow lines on the right side of the system, indicating a significantly lower velocity of fluid molecules compared to Group A and Group I. This suggests that as the torso angle of attack increases, the impact of the torso’s largest cross-section on the overall aerodynamic performance is much greater than other parts, such as the lower limbs. The flow lines between the thigh and torso exhibit more blue and green colors with an increasing torso angle of attack, indicating that the impact spreads throughout the whole system.

### Effects of torso, thigh, ankle, and hip angles

As shown in Fig. 5 of group A, G and G there exists a local optimal solution for the thigh attack angle $\:\beta\:$, where $\:\beta\:=24^\circ\:$, at which point the system experiences the minimum air resistance. The relationship between thigh angle of attack $\:\beta\:$ and system resistance is not linear, as observed in the pathlines of Group G and H. Focusing on the flow lines to the right of the hip-knee-ankle line, the color gradually becomes lighter as the thigh angle of attack increases, suggesting that the hindrance effect of the lower limbs on the fluid diminishes. This leads to more high-speed fluid molecules bypassing the lower limbs rather than exchanging energy with the system, resulting in air resistance. However, an overall view of the right-side flow lines reveals that the system’s performance declines as the torso position rises due to the increased thigh angle of attack. Therefore, a local optimal solution for the thigh angle of attack exists only when $\:\beta\:=24^\circ\:$, The optimal thigh angle minimizes interference drag between the torso and thighs by streamlining airflow along the body’s longitudinal axis.

The cumulative drag diagram (Fig. 5 of group A, E, and F) shows that increasing the ankle joint angle leads to greater aerodynamic optimization losses and higher air resistance for the multi-body system. Reducing the ankle joint angle to the minimum possible while maintaining an efficient skating posture could improve performance. Figure 6 suggests that an increased ankle joint angle directly influences the aerodynamic performance of airflow in the lower part of the multi-body system, with more light-colored flow lines indicating that the increased angle interferes with air flow, resulting in higher air resistance and negatively impacting the athlete’s speed and efficiency. The increased frontal area due to a higher ankle joint angle also contributes to greater air resistance. Comparing Group A to Groups E and F, the flow lines at the same position are dominated by relatively low-speed blue-green lines, suggesting that a lower ankle joint angle may be more beneficial for reducing air resistance.

According to Fig. 5 of group A, B, C and D, the maximum air resistance is experienced by Group D, corresponding to the hip joint abduction angle $\:\epsilon\:=4^\circ\:$. The flow lines near the hip-knee-ankle line are affected by the hip joint abduction angle. Group B has more light-colored lines in the corresponding position compared to Group A, indicating that a positive hip joint abduction angle brings negative air resistance benefits. The flow line of Group C near the hip-knee-ankle line is like Group A, with no significant difference compared to Group B. The air resistance experienced by Group C is greater than Group B, but the values are very close. Control Group D ($\:\epsilon\:=-4^\circ\:$.) was added to verify whether the improvement of negative hip joint abduction angle is inversely proportional to resistance. The flow line diagrams show significantly more light-colored lines near the knee joint in Group D, proving that a larger inward hip joint abduction angle leads to a significant decrease in aerodynamic performance, resulting in even greater air resistance. The optimal hip abduction angle narrows the athlete’s lateral profile, aligning the pelvis with the airflow to reduce cross-sectional width, thus minimizing frontal area and drag.

## Discussions

The optimal posture (Group O: $\:\alpha\:=2^\circ\:$, $\:\beta\:=24^\circ\:$, $\:\gamma\:=45^\circ\:$ ) balances minimized projected frontal area and suppression of flow separation. A slight torso angle ($\:\alpha\:=2^\circ\:$) streamlines airflow over the chest. This “sweet spot” prioritizes coherent flow over minimal frontal area principle validated in cycling and alpine skiing studies. The thigh angle ($\:\beta\:=24^\circ\:$) aligns legs with the airflow, avoiding interference drag from hip-thigh vortices, while hip abduction ($\:\epsilon\:=-2^\circ\:$) narrows the lateral profile, cutting cross-sectional width. Together, these adjustments reduce cumulative drag by 13.2% over suboptimal postures. Lower limb angles play a secondary role due to their smaller cross-sectional area. However, the ankle angle ($\:\gamma\:=45^\circ\:$) reduces ground clearance, potentially exploiting the Extreme Ground Effect (EGE), where proximity to the surface accelerates underbody airflow via Coanda adhesion, lowering pressure drag. Simulations suggest EGE may boost dynamic lift by 5–10%, though experimental validation is needed to confirm its impact during in-run phases. While ankle adjustments contribute minimally to drag reduction, their influence on ground interactions highlights the need for multi-body aerodynamic frameworks that account for surface proximity.

In practical applications, wearable sensors such as inertial measurement units (IMUs) or embedded motion capture systems could provide real-time posture feedback during training, enabling incremental adjustments. However, variability in equipment and environmental factors may modulate optimal angles—a critical area for field studies.

## Conclusion

This study presents a systematic investigation into posture-dependent aerodynamic resistance during the in-run phase of ski jumping, employing high-resolution CFD simulations and an athlete-specific model. The findings underscore the dominant influence of torso attack angle on aerodynamic performance, with the optimal range identified as $\:\alpha\:\in\:\left[0^\circ\:,2^\circ\:\right]$. While lower limb positioning, particularly the ankle joint angle ($\:\gamma\:\in\:\left[43^\circ\:,45^\circ\:\right]$) and thigh attack angle ($\:\beta\:\in\:\left[20^\circ\:,22^\circ\:\right]$), contributes to lift generation, minimizing aerodynamic drag is shown to be the primary objective for maximizing in-run velocity.

By integrating CFD-based analysis with biomechanics, this study provides a physics-driven framework for optimizing ski jumping posture, reducing reliance on empirical adjustments. The optimized posture configuration achieves a measurable reduction in cumulative air resistance (approximately 5%), demonstrating that small refinements in torso positioning have a more pronounced effect on drag reduction than adjustments in other joint angles. Although we did not quantitatively analyze the relationship between angular adjustments and final distance, as explored by Virmavirta et al.^15^, the results complement their findings, reinforcing the importance of drag minimization over lift generation during the in-run phase.

This work not only refines current understanding of in-run aerodynamics but also highlights the potential of digital twin methodologies in winter sports performance analysis. Beyond immediate applications in training, the approach used in this study offers a scalable methodology for exploring other aspects of technique and equipment design. By providing quantifiable insights into aerodynamic optimization, this research contributes to a more precise and analytically grounded approach to performance enhancement in ski jumping.

## Data Availability

Data is provided within the manuscript.
